# Draft Genome Sequence of the Butyric Acid Producer *Clostridium tyrobutyricum* Strain CIP I-776 (IFP923)

**DOI:** 10.1128/genomeA.00048-16

**Published:** 2016-03-03

**Authors:** François Wasels, Benjamin Clément, Nicolas Lopes Ferreira

**Affiliations:** Biotechnology Department, IFP Energies Nouvelles, Rueil-Malmaison, France

## Abstract

Here, we report the draft genome sequence of *Clostridium tyrobutyricum* CIP I-776 (IFP923), an efficient producer of butyric acid. The genome consists of a single chromosome of 3.19 Mb and provides useful data concerning the metabolic capacities of the strain.

## GENOME ANNOUNCEMENT

Butyric acid is a saturated four-carbon carboxylic acid used in chemical, food, perfume, pharmaceutical, and animal feed industries, and it is currently mainly produced by a chemical process. However, butyric acid can also be produced as an end product of fermentation by several *Clostridium* species (1). In order to enhance the butyrate production in these strains, genetic manipulation is mandatory, and several tools dedicated to the genus *Clostridium* have recently been developed. Another prerequisite for the successful genetic manipulation of these microorganisms is an investigation of their genetic information through genome sequencing. Among the acidogenic strains used so far, *Clostridium tyrobutyricum* showed the most promising capacity for the production of butyric acid (2). While the genome sequence of the strain ATCC 25755 was recently published (3), here, we present the draft genome sequence of strain CIP I-776 (IFP923), which has been shown to have the highest titers in terms of butyric acid production among a panel of wild-type strains belonging to the *Clostridium* genus (4).

Genomic DNA was extracted using the GenElute bacterial genomic DNA kit (Sigma-Aldrich) and sequenced on an Illumina MiSeq with a 2 × 250-bp paired-end sequencing kit. A total of 3,756,334 reads were assembled using the Velvet assembler (5) into 139 contigs, with a total size of 3,190,249 bp, providing 294× coverage. The average contig length was 22,951 bp, with the largest contig being 252,918 bp. The average G+C content was of 30.8%. Functional annotation was carried out using tools of the MicroScope platform (6).

Consistent with data obtained for *C. tyrobutyricum* ATCC 25755, genome analysis of strain CIP I-776 did not allow the identification of genes coding for a phosphate butyryltransferase or a butyrate kinase, which are involved in butyrate formation in other *Clostridium* species, such as *Clostridium acetobutylicum* (7). In contrast, several genes were predicted to code for enzymes that could be involved in recently identified alternative pathways (8).

While one *ack* gene coding for an acetate kinase was identified, two putative *ycf* genes coding for acyl-coenzyme A (CoA):acetoacyl-CoA transferase were detected. These genes do not have homologues in *C. acetobutylicum* and may have a key role in the conversion of butyryl-CoA to butyrate in *C. tyrobutyricum*, consistent with observations made by other authors (9). Further studies are required to investigate this alternative butyrate formation pathway, and the genomic data obtained in this study will allow the performance of efficient genetic strategies to confirm this hypothesis and increase the production capacities of the strain.

### Nucleotide sequence accession numbers.

This whole-genome shotgun project has been deposited at DDBJ/EMBL/GenBank under the accession no. FAXL00000000. The version described in this paper is the first version, FAXL01000000.
